# Supplementary material for: Development of a simple screening tool for determining cognitive status in Alzheimer’s disease
Source: PLoS One. 2023 Jan 12;18(1):e0280178. doi: 10.1371/journal.pone.0280178 (PMC9836308; doi:10.1371/journal.pone.0280178)
Supplement: Attachment — Submitted filename: Response to reviewer_1123.docx [file pone.0280178.s001.docx]

**PLOS ONE**

Referee Report on the Revised Version of Manuscript PONE-D-22-23424

“Development of a Simple Screening Tool for Determining Cognitive Status in Alzheimer’s Disease”

We are grateful for your invaluable assistance in the revision of this paper. We have closely followed the guidance provided by the reviewers and editors in this revision. Detailed responses to the comments are provided below.

**Journal Requirements**

1. Please ensure that your manuscript meets PLOS ONE's style requirements, including those for file naming. The PLOS ONE style templates can be found at
   <https://journals.plos.org/plosone/s/file?id=wjVg/PLOSOne_formatting_sample_main_body.pdf> and 
   <https://journals.plos.org/plosone/s/file?id=ba62/PLOSOne_formatting_sample_title_authors_affiliations.pdf>

**Reply:**

The formatting of the revised manuscript has been adjusted in accordance with journal requirements.

2. Please provide additional details regarding participant consent. In the ethics statement in the Methods and online submission information, please ensure that you have specified (1) whether consent was informed and (2) what type you obtained (for instance, written or verbal, and if verbal, how it was documented and witnessed). If your study included minors, state whether you obtained consent from parents or guardians. If the need for consent was waived by the ethics committee, please include this information. If you are reporting a retrospective study of medical records or archived samples, please ensure that you have discussed whether all data were fully anonymized before you accessed them and/or whether the IRB or ethics committee waived the requirement for informed consent. If patients provided informed written consent to have data from their medical records used in research, please include this information. Once you have amended this/these statement(s) in the Methods section of the manuscript, please add the same text to the “Ethics Statement” field of the submission form (via “Edit Submission”). For additional information about PLOS ONE ethical requirements for human subjects research, please refer to <http://journals.plos.org/plosone/s/submission-guidelines#loc-human-subjects-research>.

**Reply:**

As the data were analyzed retrospectively and anonymously from a database established in previous Taiwanese studies, informed consent was waived. We have provided the information in the Methods section of the revised manuscript (Lines 12 to 15, Page 7).

3. We note that you have indicated that data from this study are available upon request. PLOS only allows data to be available upon request if there are legal or ethical restrictions on sharing data publicly. For more information on unacceptable data access restrictions, please see http://journals.plos.org/plosone/s/data-availability#loc-unacceptable-data-access-restrictions.

In your revised cover letter, please address the following prompts:

a) If there are ethical or legal restrictions on sharing a de-identified data set, please explain them in detail (e.g., data contain potentially sensitive information, data are owned by a third-party organization, etc.) and who has imposed them (e.g., an ethics committee). Please also provide contact information for a data access committee, ethics committee, or other institutional body to which data requests may be sent.

b) If there are no restrictions, please upload the minimal anonymized data set necessary to replicate your study findings as either Supporting Information files or to a stable, public repository and provide us with the relevant URLs, DOIs, or accession numbers. For a list of acceptable repositories, please see http://journals.plos.org/plosone/s/data-availability#loc-recommended-repositories.

We will update your Data Availability statement on your behalf to reflect the information you provide.

**Reply:**

We have uploaded a de-identifying minimum dataset that can support the conclusions of the manuscript on BioStudies Submission Tool (accession number: S-BSST951). We have added a section (Data Availability) in the revised manuscript (Lines 14 to 15, Page 24).

4. Please review your reference list to ensure that it is complete and correct. If you have cited papers that have been retracted, please include the rationale for doing so in the manuscript text, or remove these references and replace them with relevant current references. Any changes to the reference list should be mentioned in the rebuttal letter that accompanies your revised manuscript. If you need to cite a retracted article, indicate the article’s retracted status in the References list and also include a citation and full reference for the retraction notice.

**Reply:**

We have corrected and updated the reference list in the revised manuscript (Page 25 to 35).

5. Additional Editor Comments: I agree with both reviewers for a minor revision of the manuscript and I think they raised interesting points to improve the quality of your manuscript.

**Reply:**

We have closely followed the guidance provided by the reviewers in this revision.

**Reviewer 1**

Authors propose an interesting and easy-to-administer tool for the detection of cognitive impairment and, even more interestingly, for differentiating between MCI and dementia. I certainly encourage this paper to be published because of its likely utility in daily clinical practice.

1. I only have one minor comment that I would like the authors may discuss. Although they mentioned educational level and, as a matter of fact, they did not find an association with the pathology evolution, in such a study, I would expect some considerations about cognitive reserve and its role in modulating not only the onset, but also the progression of cognitive impairment.

**Reply:**

We have addressed the issue of cognitive reserve in the Discussion section of the revised manuscript (Lines 10 to 14, Page 14 and Line 20, Page 21 to Line 1, Page 22).

2. Furthermore, although MMSE has important limitations (clinical, first of all!), it would be helpful also a comparison/a critical consideration or comment referring HAI-SAC to MMSE which is, at the date, the most applied screening test for dementia (authors certainly know that MoCA is more difficult and more focused on executive functions rather than MMSE which is easier and, for some aspects, more suitable for older individuals).

**Reply:**

We have added a comparison and addressed the sensitivity and specificity of HAI-SAC and MMSE in the Discussion section of the revised manuscript (Lines 11 to 18, Page 22). We have also mentioned the limitation imposed by our use of an extracted MMSE score (Lines 21 to 23, Page 23).

**Reviewer 2**

1. ABSTRACT should be re-written in order to provide clearer information to the readers.

**Reply:**

We have re-written the Abstract in the revised manuscript (Page 3).

2. Please provide the mean age and female percentage of the study population.

**Reply:**

We have provided this information in the Results (Lines 12 to 14, Page 13) and Abstract (Lines 5 to 6, Page 3) section of the revised manuscript.

3. How many patients were diagnosed with MCI, AD and control group?

**Reply:**

A total of 397 participants were diagnosed with AD dementia, 231 were diagnosed with MCI, and 136 were diagnosed as CU. This information has been included in the Methods section (Lines 10 to 12, Page 7) and Table 1 of the revised manuscript. We have also provided this information in the Abstract section of the revised manuscript (Lines 6 to 7, Page 3).

4. What was the sensitivity, specificity of the HAI-SAC to detect MCI and AD?

**Reply:**

In differentiating between MCI and NC, the sensitivity of HAI-SAC was 0.87 and the specificity was 0.58, with a cut-off score of 41/42. In differentiating between MCI and DAT, the sensitivity of HAI-SAC was 0.78 and the specificity was 0.84, with a cut-off score of 33/34. In differentiating between NC and DAT, the sensitivity of HAI-SAC was 0.99 and the specificity was 0.89, with a cut-off score of 35/36. This information has been included in the Results section of the revised manuscript (Lines 2 to 11, Page 16; Lines 7 to 14 and Lines 18 to 21, Page 17). We have also provided the information in the Abstract section of the revised manuscript (Line 19, Page 3 to Line 1, Page 4) and corrected typos in Table 4 (Page 19).

5. And it would be better if the authors mention the AUC results about discriminating MCI from NC/AD.

**Reply:**

This information has been included in the Results section and Table 4 of the revised manuscript (Pages 18 to 19). We have also included this information in the Abstract section of the revised manuscript (Line 22, Page 3 to Line 1, Page 4).

6. INTRO: The authors should give some examples about short screening tools (<https://pubmed.ncbi.nlm.nih.gov/28660847/>, <https://pubmed.ncbi.nlm.nih.gov/29923472/>)

**Reply:**

We have included an introduction to these tools in the Introduction section of the revised manuscript (Lines 13 to 15, Page 5).

7. RESULTS is well-written, but is there any information about comorbidities of the patients?

**Reply:**

We have listed the proportion of individuals with hypertension, diabetes, hypercholesterolemia, or coronary artery disease across groups in the Methods (Lines 6 to 8, Page 7) and Results (Lines 1 to 4, Page 14) sections and Table 1 of the revised manuscript (Page 14).
